# Supplementary figures and images for: Virseqimprover: an integrated pipeline for viral contig error correction, extension, and annotation
Source: PeerJ. 2025 Jan 10;13:e18515. doi: 10.7717/peerj.18515 (PMC11727651; doi:10.7717/peerj.18515)

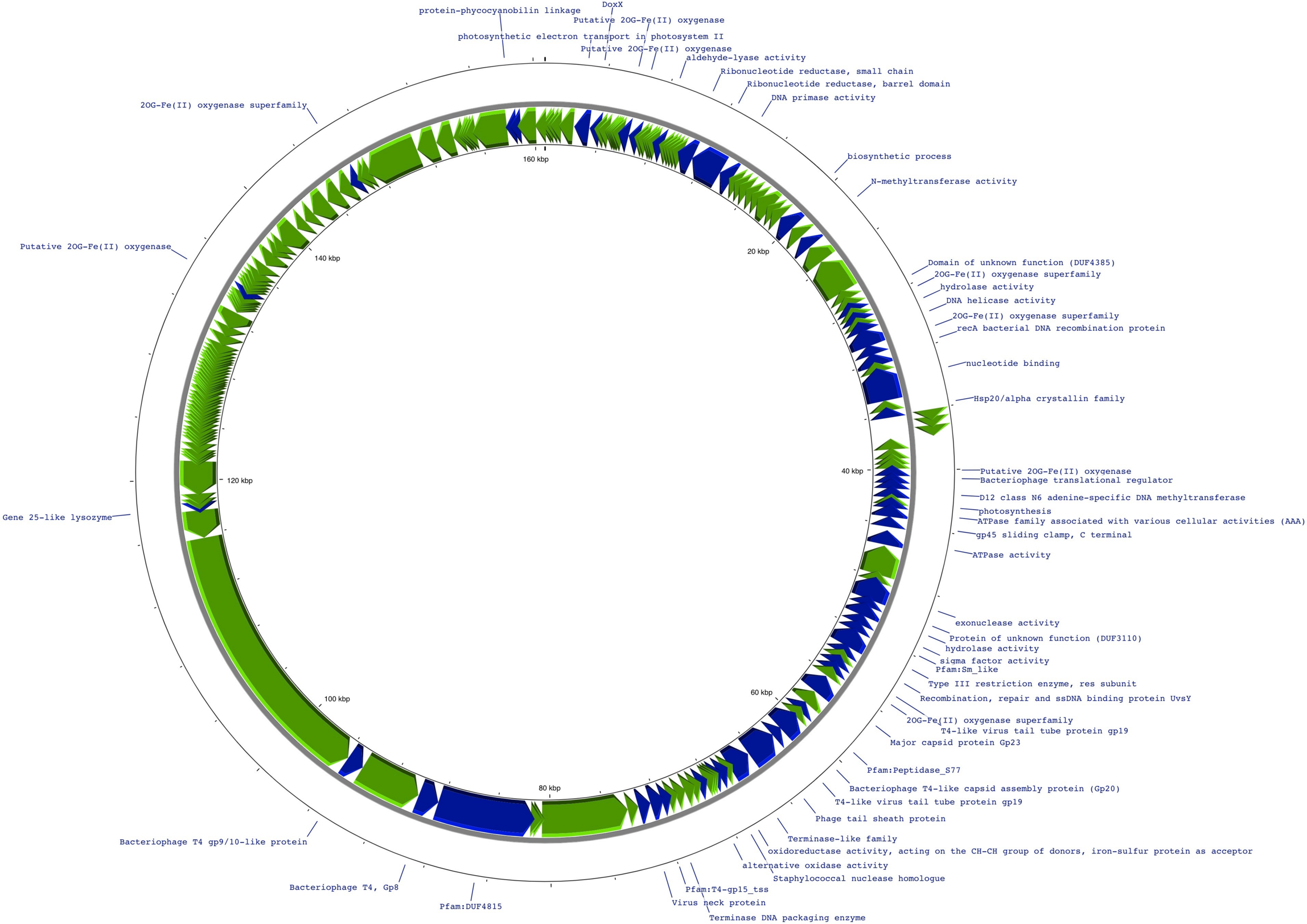

Supplement: Supplemental Information 1 — The blue arrows are annotated proteins while the green arrows are unannotated proteins. [file peerj-13-18515-s001.pdf]

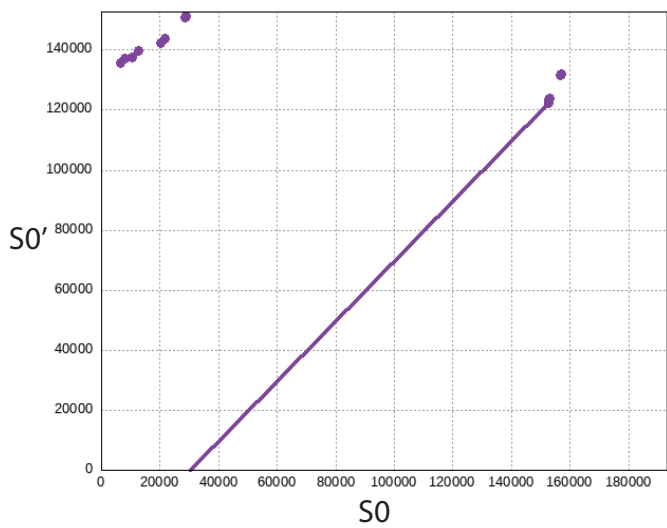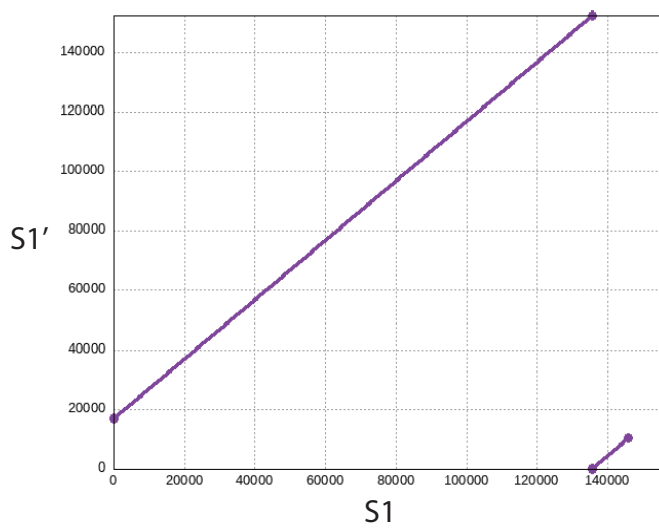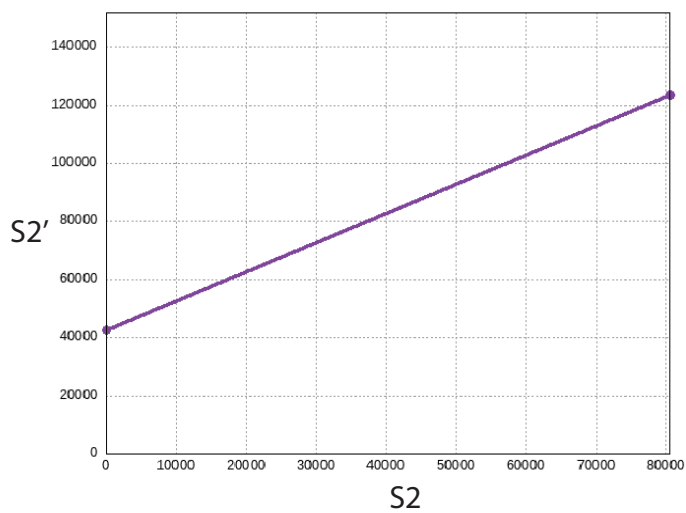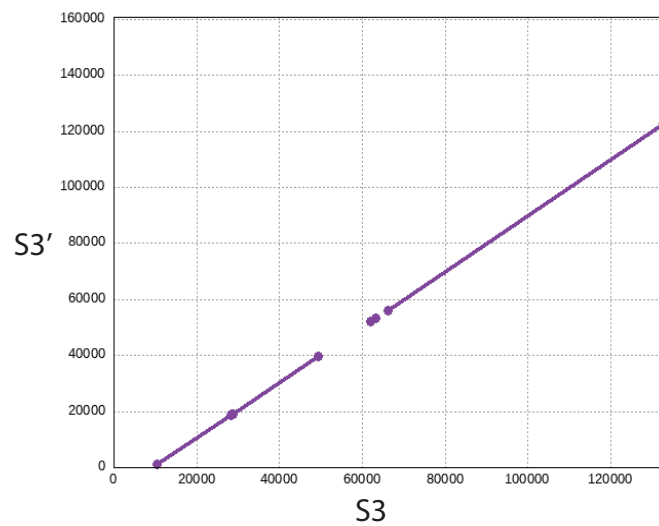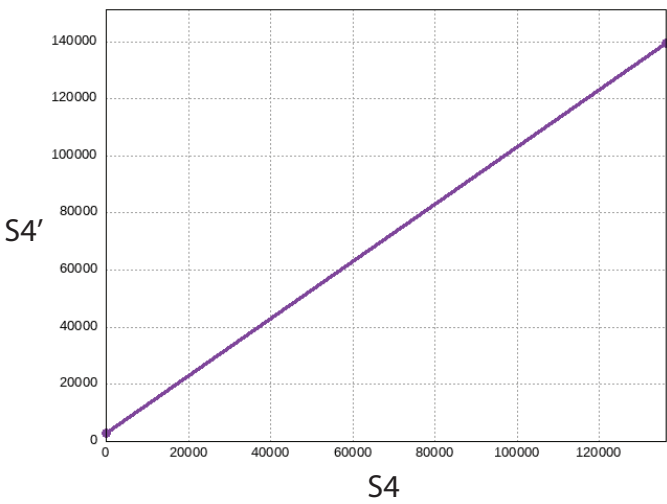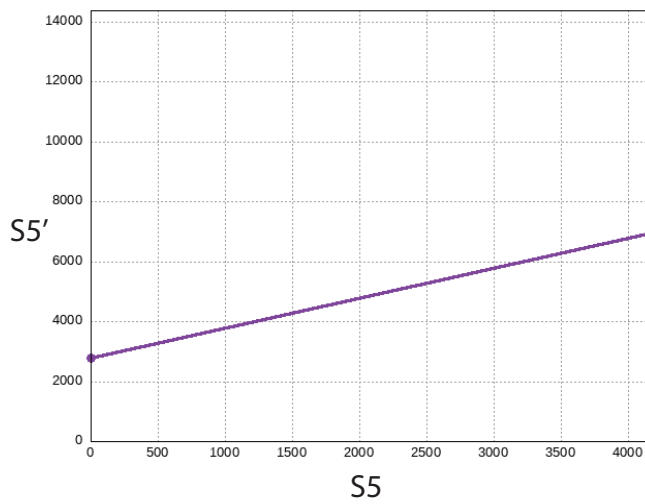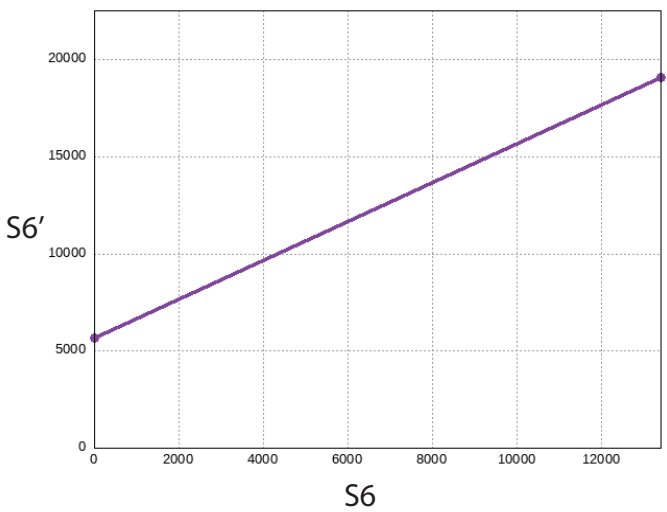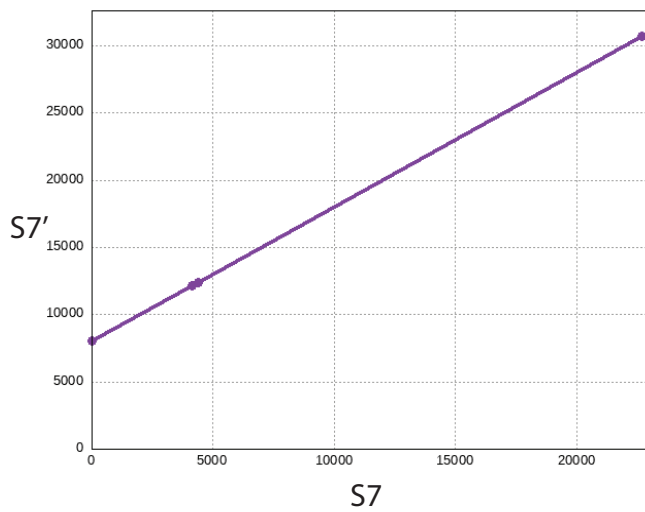

Supplement: Supplemental Information 2 [file peerj-13-18515-s002.pdf]
